# Overview of systematic reviews on the health-related effects of government tobacco control policies

**DOI:** 10.1186/s12889-015-2041-6

**Published:** 2015-08-05

**Authors:** Steven J. Hoffman, Charlie Tan

**Affiliations:** Global Strategy Lab, Faculty of Law, University of Ottawa, 57 Louis Pasteur Street, Ottawa, K1N 6N5 ON Canada; Department of Clinical Epidemiology & Biostatistics and McMaster Health Forum, McMaster University, Hamilton, ON Canada; Department of Global Health & Population, Harvard T.H. Chan School of Public Health, Harvard University, Boston, MA USA; Michael G. DeGroote School of Medicine, McMaster University, Hamilton, ON Canada

## Abstract

**Background:**

Government interventions are critical to addressing the global tobacco epidemic, a major public health problem that continues to deepen. We systematically synthesize research evidence on the effectiveness of government tobacco control policies promoted by the Framework Convention on Tobacco Control (FCTC), supporting the implementation of this international treaty on the tenth anniversary of it entering into force.

**Methods:**

An overview of systematic reviews was prepared through systematic searches of five electronic databases, published up to March 2014. Additional reviews were retrieved from monthly updates until August 2014, consultations with tobacco control experts and a targeted search for reviews on mass media interventions. Reviews were assessed according to predefined inclusion criteria, and ratings of methodological quality were either extracted from source databases or independently scored.

**Results:**

Of 612 reviews retrieved, 45 reviews met the inclusion criteria and 14 more were identified from monthly updates, expert consultations and a targeted search, resulting in 59 included reviews summarizing over 1150 primary studies. The 38 strong and moderate quality reviews published since 2000 were prioritized in the qualitative synthesis. Protecting people from tobacco smoke was the most strongly supported government intervention, with smoke-free policies associated with decreased smoking behaviour, secondhand smoke exposure and adverse health outcomes. Raising taxes on tobacco products also consistently demonstrated reductions in smoking behaviour. Tobacco product packaging interventions and anti-tobacco mass media campaigns may decrease smoking behaviour, with the latter likely an important part of larger multicomponent programs. Financial interventions for smoking cessation are most effective when targeted at smokers to reduce the cost of cessation products, but incentivizing quitting may be effective as well. Although the findings for bans on tobacco advertising were inconclusive, other evidence suggests they remain an important intervention.

**Conclusion:**

When designing and implementing tobacco control programs, governments should prioritize smoking bans and price increases of tobacco products followed by other interventions. Additional studies are needed on the various factors that can influence a policy’s effectiveness and feasibility such as cost, local context, political barriers and implementation strategies.

**Electronic supplementary material:**

The online version of this article (doi:10.1186/s12889-015-2041-6) contains supplementary material, which is available to authorized users.

## Background

The global tobacco epidemic is a major public health problem that continues to deepen, with nearly 1 billion smokers worldwide in 2012 [1]. It is the leading cause of preventable death, resulting in approximately 6 million unnecessary deaths per year [2, 3]. Cigarettes will kill half their current users through conditions such as cardiovascular disease, respiratory conditions and cancers, and mortality from smoking is expected to increase to an estimated 8 million people per year by 2030 [3]. Secondhand smoke (SHS) is a significant concern as well: more than 600,000 of the total annual deaths from smoking are due to SHS exposure [2].

The year 2015 marks the tenth anniversary since the World Health Organization (WHO) Framework Convention on Tobacco Control (FCTC) came into force, the first international treaty specially adopted through WHO. This instrument aims to reduce tobacco consumption and protect all people from tobacco exposure through provisions that direct countries to implement tobacco control programs [4]. To guide countries in this process, WHO introduced the six MPOWER measures (Table 1), each of which corresponds to one or more FCTC provisions [5]. The 180 parties to the FCTC (as of May 2015) are all mandated to take action [6]. However, the bi-annual reports that ratifying countries must submit show uneven progress [7].

**Table 1:** MPOWER measures

MPOWER measure	Description
Monitor tobacco use and prevention policies	Surveillance of the prevalence, determinants and impacts of tobacco use, and measuring the effects of tobacco control interventions (FCTC Article 20)
Protect people from tobacco smoke	Reduce secondhand smoke exposure through comprehensive smoke-free legislation in public spaces, including all indoor workplaces (FCTC Article 8)
Offer help to quit tobacco use	Cessation support through advice from healthcare providers, telephone quit lines and easily-accessible or low cost medications (FCTC Article 14)
Warn about the dangers of tobacco	Warnings on tobacco packaging and anti-tobacco media campaigns to promote awareness on the health consequences of smoking (FCTC Article 11 and Article 12)
Enforce bans on tobacco advertising and sponsorship	Bans on direct (e.g., television advertisements, billboards) and indirect (e.g., industry-sponsored events) marketing of tobacco products (FCTC Article 13)
Raise taxes on tobacco	Increasing the price of tobacco products through taxation (FCTC Article 6)

The aim of this systematic overview of systematic reviews is to take stock of the global research evidence base in this decennial year about the likely health effects of different government tobacco control policies. This provides policymakers with an evidence-based resource to help with policy deliberations and decision-making on setting priorities for national FCTC implementation [8]. Systematic reviews aim to identify, assess and synthesize all available primary evidence on a research question, and use an objective, optimized and structured methodology to maximize transparency and minimize bias. Overviews of systematic reviews build on the strengths of individual reviews and add breadth by integrating the findings of many reviews together.

## Methods

Relevant systematic reviews were identified through systematic searches of the electronic databases *Health Evidence* for research on public health and health promotion, *Health Systems Evidence* for research on health systems, *Rx for Change* for research on behaviour-change interventions, *Cochrane Database of Systematic Reviews* for research on healthcare and health policy, and *Database of Abstracts of Reviews of Effects* for research on healthcare and health services delivery, published up to March 2014. These databases are continuously updated and together represent the leading repositories of health-related systematic reviews. For *Health Evidence*, the search term “tobacco use” was entered; for other databases, “tobacco” was entered. This search strategy was designed to be very broad and capture as many systematic reviews related to tobacco use as possible. Relevant reviews in monthly updates from *Health Evidence, Health Systems Evidence* and the *Quebec Public Health Research Network* were included as well up to August 2014. Two tobacco control experts known to the authors were also consulted to identify any reviews missed by the systematic searches. An additional targeted search for systematic reviews on mass media campaigns was conducted in May 2015 in response to peer review feedback.

The present overview includes systematic reviews evaluating government tobacco control policies. Reviews that did not follow a systematic methodology and other overviews of systematic reviews were not included; the latter’s references were scanned to identify reviews for inclusion. The MPOWER components were used as an organizing framework to classify the interventions. Clinical interventions, such as pharmacologic therapy and counselling services, were not included. Reviews on multicomponent programs were excluded if the effects of individual interventions were not independently studied or could not be differentiated. Outcome measures relating to tobacco use, SHS exposure and primary health outcomes were considered. Tobacco use outcomes include smoking prevalence, quantity of cigarettes consumed, smoking cessation and smoking initiation. SHS exposure outcomes include both self-reported and biomarker-validated measures of exposure. Health outcomes include hospital admissions and risk of any adverse medical event. Measures that did not directly assess change in smokers, such as recall of media campaigns and perceptions of smoking, were not included. Reviews were not excluded on the basis of language.

Titles and abstracts were assessed to identify reviews for inclusion. The full texts were then read to confirm inclusion. The following data from selected studies were abstracted into a summary table: number of studies included, year of last search and key findings. The quality ratings given to each review by their source databases were extracted as well. *Health Evidence* uses an independent quality assessment tool specific for public health intervention literature [9]. Total scores can range from 0 to 10, based on which a quality rating is assigned: “strong” (8 to 10), “moderate” (5 to 7) or “weak” (4 or less). *Health Systems Evidence* and *Rx for Change* use the AMSTAR tool, which evaluates the quality of systematic reviews and meta-analyses by 11 quality criteria [10–12]. Reviews with scores from 9 to 11 are rated as “high,” from 5 to 8 as “medium,” and from 0 to 4 as “low.” For this overview, the “strong,” “moderate” and “weak” terminology was used for AMSTAR to maintain consistency with *Health Evidence. Cochrane Database of Systematic Reviews* does not provide ratings and *Database of Abstracts of Reviews of Effects* offers a descriptive quality assessment [13]. A quality assessment was independently performed using the AMSTAR tool for reviews without a numerical quality rating. Strong and moderate quality reviews published since 2000 were prioritized in the qualitative synthesis.

## Results

**Fig. 1:**
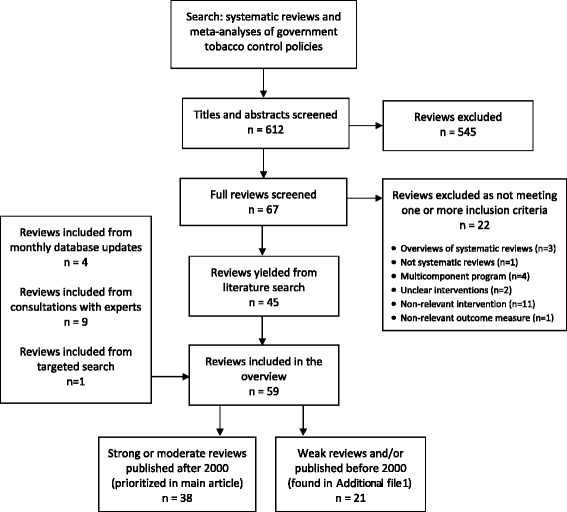
Process of review selection

**Fig. 2:**
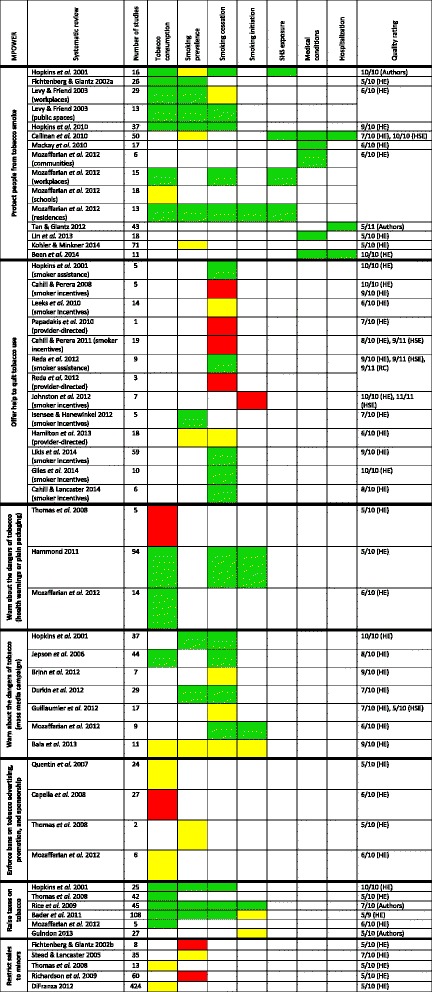
Overview of results from strong and moderate quality systematic reviews published since 2000. A green square indicates the review found the intervention had a beneficial effect on the outcome measure. A yellow square indicates the review found an unclear or conditional effect. A red square indicates the review found the intervention had no effect. Thomas *et al*. 2008 could not be included in this table under “Protect people from tobacco smoke” since it looked at the effect of smoking bans on social inequalities in smoking; it did not evaluate their overall impact or the conditions under which they are effective. Kohler & Minkner 2014 conducted a review of state smoke-free laws and petitions, initiatives and referendums to protect non-smokers in Germany, finding 56 laws and amendments and 15 initiatives. HE = *Health Evidence*; HSE = *Health Systems Evidence*; RC = *Rx for Change*

Six hundred and twelve reviews were identified through the search strategy. Screening titles and abstracts yielded 67 reviews that possibly met the inclusion criteria. Two reviewers screened the full-text of reviews and confirmed 45 met the inclusion criteria. Any conflicts were resolved by discussion between the two reviewers and a third reviewer was consulted when required. Four additional reviews for inclusion were identified from monthly updates, nine were identified by tobacco control experts and one was identified from the targeted search for reviews on mass media campaigns. In total, 59 reviews were included that together summarized over 1150 primary studies (see Fig. 1) [14–72].^1^ The 22 reviews excluded after full-text screening either evaluated excluded outcome measures (*n* = 1) [73], were overviews of systematic reviews (*n* = 3) [74–76], did not follow a systematic methodology (*n* = 1) [77], only reported results of multicomponent programs (*n* = 4) [78–81], did not adequately describe what interventions were evaluated (*n* = 2) [82, 83], or were not relevant for assessing government tobacco control policies (*n* = 11) [84–94]. Findings from the 38 reviews that were either strong or moderate quality and published since 2000 are presented here (see Fig. 2) [14–51]. A Web Appendix contains individualized summaries of these reviews as well as the lower quality and older reviews (see Additional file 1) [52–72].

### Protect people from tobacco smoke

Twelve reviews summarize the health-related effects of smoking bans and restrictions in public spaces, workplaces or residences. Three are strong in quality [14–16], eight are moderate [17–24] and one is rated as moderate by HE and as strong by HSE [25]. Overall, most of the eight reviews on smoking behaviour reported reductions in smoking prevalence and cigarette consumption and increases in smoking cessation [14, 15, 17–19, 21, 24, 25]. Hopkins et al. found that workplace smoke-free policies lead to an absolute reduction in smoking prevalence of 3.4 % (interquartile range (IQR) = −6.3 to −1.4), reduced cigarette consumption by 2.2 cigarettes per day (IQR = −1.7 to −3.3), increased quit attempts by 4.1 % (IQR = −0.7 to 6.8) and increased successful cessation by 6.4 % (IQR = 2.0 to 9.7) [15]. Three reviews found inconsistent evidence for smoking prevalence or cessation, but reported improvements in other smoking behaviour outcomes [14, 18, 21]. Thomas et al. looked at the effect of smoking bans on social inequalities in smoking [19] and Kohler and Minkner reviewed the conditions under which state smoking bans are effective [24], but neither assessed their overall efficacy. Three reviews investigating SHS all reported reductions in SHS exposure with smoke-free policies, in both adults and children and across various settings including workplaces, public spaces and hospitality establishments [14, 21, 25]. Similarly, all six reviews investigating primary health outcomes found decreases in adverse events [16, 20–23, 25]. Tan and Glantz looked at hospital admissions data and found a reduced risk of admission for coronary events (relative risk (RR) = 0.85, 95 % confidence interval (CI) = 0.82 to 0.88), other heart diseases (RR = 0.61, 95 % CI = 0.44 to 0.85), cerebrovascular accidents (RR = 0.81, 95 % CI = 0.70 to 0.94) and respiratory diseases (RR = 0.76, 95 % CI = 0.68 to 0.85) with smoke-free policies [22]. The reductions were greatest with comprehensive policies that banned smoking in workplaces, restaurants and bars. Therefore, there is very strong evidence that smoke-free legislation reduces smoking behaviour, exposure to SHS and adverse health outcomes.

### Offer help to quit tobacco use

Twelve reviews present research evidence on the effect of financial assistance or incentives for smokers to quit smoking and for healthcare professionals to provide smoking cessation interventions.^2^ Eight are strong in quality [14, 26–32] and four are moderate [33–36]. Financial interventions appear to have different effects depending on whether they are incentives to quit smoking or assistance to lower the cost of cessation therapies, and whether they target smokers or healthcare providers. Two reviews investigated smoker-directed financial assistance and found increased uptake of cessation therapies and greater levels of smoking cessation [14, 28]. The effect of incentives and competitions for smokers is less clear, with four reviews reporting increased cessation [30–32, 35] and four reviews finding an unclear or no effect [26, 27, 29, 33]. Giles et al. [31] and Cahill and Lancaster [32] found that smokers offered incentives were 2.48 times (95 % CI = 1.77 to 3.46) and 1.60 times (95 % CI = 1.12 to 2.30) more likely to quit smoking, respectively. In pregnant women, Likis et al. reported that financial incentives increased smoking cessation and were the most important component of multicomponent programs that promote cessation [30]. Conversely, Cahill and Perera found no effect of incentives on long-term quit rates [27], and a separate review by the same authors estimated that community “quit-and-win” contests lead to fewer than one in 500 smokers quitting smoking [26]. The three reviews that looked at provider-directed financial interventions found no effect on smoking abstinence and prevalence in patients [28, 34, 36]. Financial interventions may thus be more effective for smoking cessation when they are targeted to make cessation therapies more affordable, but incentivizing quitting may be effective as well. They do not appear to influence smoking behaviour when directed at healthcare professionals.

### Warn about the dangers of tobacco

Three reviews contained evidence on the effects of health warning labels or plain packaging on tobacco products. All three are moderate in quality [19, 21, 37]. Two reviews found health warning labels decrease smoking behaviour, reporting reductions in tobacco use and increases in motivation to quit, quitting likelihood and likelihood of abstinence after quitting [21, 37]. Thomas et al. found no effect [19]. Among seven reviews assessing mass media campaigns, four are strong in quality [14, 38–40] and three are moderate [21, 41, 42]. Five reviews looked at media campaigns as part of comprehensive tobacco control programs and four reported reductions in smoking behaviour [14, 21, 38, 41]. Bala et al. showed inconsistent effects and raised concerns about the quality of the evidence [40]. An update to this review found strong evidence that mass media campaigns, within the context of multicomponent programs, promote cessation and reduce smoking prevalence [41]. This updated review identified several features of effective campaigns: wide population reach, high intensity, long duration, use of television and messages on the negative health effects of smoking [41]. The remaining systematic reviews focused on mass media campaigns in subpopulations. Guillaumier et al. found insufficient evidence for their effect on smokers of low socioeconomic status [42], while Brinn et al. [39] reported inconsistent evidence for their effect on young people. Therefore, the evidence suggests that interventions cautioning people about tobacco’s harms may be effective strategies, with mass media campaigns being potentially important parts of multicomponent programs.

### Enforce bans on tobacco advertising, promotion and sponsorship

Four reviews present research evidence on the health-related effects of tobacco advertising bans and restrictions, all of which are moderate in quality [19, 21, 43, 44]. None of the reviews found clear reductions in smoking behaviour, with Quentin et al. reporting inconsistent effects [43] and Capella et al. showing no reduction in cigarette consumption [44]. Although Mozaffarian et al. did not report any direct effects, this review postulated that advertising restrictions should decrease smoking behaviour based on the well-established association between advertisements and tobacco use [21]. Indeed, individual studies have demonstrated that tobacco marketing and cigarette use in movies alter adolescents’ attitudes and susceptibilities to smoking, which in turn places them at risk of initiating cigarette use and continuing this behaviour into adulthood [95–99]. Although systematic reviews have not conclusively shown that banning tobacco marketing is an effective tobacco control measure, substantial evidence exists on the harmful consequences of unregulated advertising on smoking behaviour.

In addition, there are individual studies that support the effectiveness of these interventions. Saffer and Chaloupka looked at 22 OECD countries and showed that comprehensive advertising bans reduce tobacco consumption, but that limited advertising bans—where other avenues of promotion are still available to companies—do not reduce tobacco consumption [100]. An analogous study by Blecher that included the OECD countries as well as 30 developing countries had similar findings [101]. A sub-analysis of the developing countries showed that both comprehensive and limited bans reduced smoking in these countries, with comprehensive bans having greater effects [101]. The scope of advertising restrictions and how they are enforced may therefore be critical factors in their effectiveness.

### Raise taxes on tobacco

Among the six reviews evaluating tobacco price increases, one is high in quality [14] and five are moderate [19, 21, 45–47]. All but one of the reviews found that increasing the price of tobacco reduces smoking behaviour, with decreases in cigarette consumption and smoking prevalence and increases in smoking cessation. Hopkins et al. combined seven studies that estimated price elasticities of demand and found that every 10 % increase in cigarette prices decreased smoking prevalence and cigarette consumption by 3.7 and 2.3 %, respectively [14]. Price increases appear to be most effective among adolescents, young adults and persons of low socioeconomic status [19, 45, 46]. Rice et al. reported negative price elasticities for smoking participation, prevalence, consumption and initiation, and greater smoking cessation in youths with price increases [46]. However, Guindon found inconclusive results on the effect of tobacco price increases on smoking initiation [47]. Overall the evidence indicates increasing the price of tobacco products is a very important intervention for tobacco control.

### Restrict sales to minors

Five reviews summarize the health-related effects of restricting youth access to tobacco products, all of which are moderate in quality [19, 48–51]. This intervention is not part of MPOWER but is promoted in the FCTC under Article 16 (“prohibiting sales to and by minors”). Across the systematic reviews there was no consistent effect found for youth access interventions on smoking behaviour. Fichtenberg and Glantz showed that such restrictions do not reduce smoking prevalence among teens, even with high compliance among cigarette merchants [48]. However, DiFranza reported that banning the sale of tobacco products to minors is effective in decreasing youth smoking, but only if the restrictions are strongly enforced [51]. The three remaining reviews found inconsistent evidence [19, 49, 50]. The literature on youth access interventions is thus inconclusive; restrictions, if they are effective, appear to depend on robust enforcement.

## Discussion

### Principal findings

This systematic overview of systematic reviews summarizes the research evidence on the likely health-related effects of government tobacco control policies promoted in the FCTC, identifying gaps in the literature and providing a framework for policy deliberations and future research. It includes 59 systematic reviews, encompassing more than 1150 individual studies.

Among the government tobacco control policies identified, protecting people from tobacco smoke through smoking bans and price increases of tobacco products have the strongest evidence of effectiveness. Smoke-free policies were consistently associated with reductions in smoking behaviour, SHS exposure and adverse health outcomes. Their success has been demonstrated in diverse settings. Robust evidence was similarly found for raising tobacco prices, such as through taxation, with decreases in smoking behaviour found by five reviews. The cumulative evidence from this overview suggests that smoke-free policies and tobacco taxation may be the two most important interventions promoted by the FCTC and should be prioritized by governments when developing their tobacco control programs.

Positive results were also found for warning people about the dangers of tobacco through mass media campaigns and cigarette packaging interventions, with the former likely being integral parts of multicomponent programs. Financial assistance reducing the costs of smoking cessation interventions and financial incentives may be effective strategies; the effect of provider-directed interventions is less clear. Limited evidence was found for advertising restrictions, but evidence on how advertisements influence smoking behaviour, the substantial investments that cigarette companies have made to promote their products and individual studies demonstrating their effectiveness all suggest these interventions remain important. No reviews were identified for monitoring tobacco use and prevention policies, indicating a need for research syntheses on their effectiveness.

Although this overview assesses the effectiveness of individual government tobacco control policies, countries should implement comprehensive programs that contain two or more FCTC provisions and that are tailored specifically to their national situations. Such multicomponent programs are the most effective approach to tobacco control, with policies acting synergistically when enacted together [102, 103]. While greater research evidence exists for certain interventions, all tobacco control measures under the FCTC are important and should ideally be implemented concurrently. However, it is often not possible for countries to dedicate equal resources to each policy or to introduce them simultaneously. The 2014 Global Progress Report showed variability in implementation rates across FCTC provisions, with over 90 % of parties reporting to have prioritized one or more areas of the treaty over others [104]. Lack of financial, human, research and political resources were also identified as barriers to full implementation [104]. Thus, while countries would ideally implement all tobacco control policies concurrently, regulations that ban smoking and increase tobacco product prices should be prioritized in situations where resources are constrained. The other FCTC provisions can then further strengthen and work synergistically with these priority policies. This will help promote an evidence-based prioritization of policies and maximize the likelihood that limited resources will be allocated to those measures most likely to protect people from the harmful effects of tobacco.

### Strengths and limitations

This overview has several methodological strengths. First, prioritizing systematic reviews allowed a huge body of research evidence to be synthesized—over 1150 studies—covering a wide variety of interventions, outcomes, conditions and populations in a single analysis. Second, a systematic and transparent search protocol was implemented, minimizing bias while ensuring that as many systematic reviews evaluating government tobacco control policies as possible were identified and assessed for inclusion. Third, the quality of each systematic review was considered, with quality ratings either extracted from source databases or independently scored. These quality ratings were then used, along with the date of publication, to prioritize included reviews, maximizing confidence in the resulting analyses as a good starting point for decision-making.

This overview also has several limitations. First, searches were restricted to the five leading databases of systematic reviews; unpublished reviews will have been missed. Furthermore, many disciplines have different approaches to literature reviews that place less emphasis on describing the search strategy and inclusion/exclusion criteria. As such, some excellent comprehensive reviews may have been excluded by the databases or have low quality ratings [67, 103, 105]. Second, the intricacies and details of each individual study are invariably lost when integrating findings from over 1150 primary sources. Third, evaluations of multicomponent policies were excluded, limiting this overview to summarizing the impact of interventions when used in isolation.

### Future directions

Based on the cumulative evidence, the effectiveness of smoke-free policies is well established; the current evidence demonstrates smoke-free policies are critical tobacco control interventions. Tobacco price increases are also well supported by the literature, although future studies and reviews could helpfully assess outcomes other than smoking behaviour. More evaluations of the remaining interventions are important to determine their overall effectiveness and the conditions under which they are most impactful. For all interventions, studies and syntheses of the various factors that can influence their effectiveness and feasibility, such as cost, local context, political barriers and implementation strategies, are needed. The differential effect of these policies, such as by a nation’s level of development or by a population’s demographic characteristics, is also an important research gap. Studies and syntheses evaluating the synergistic effects of multicomponent programs and determining which combinations of policies are most effective would be particularly beneficial for governments in establishing or expanding evidence-based tobacco control programs. Research should also not be limited to the FCTC provisions; innovative and radical approaches, or “endgame proposals,” should be encouraged to further reduce and ultimately eliminate tobacco use [106].

## Conclusion

This overview of systematic reviews provides a summary of current research evidence on the effectiveness of individual government tobacco control policies. The 180 state parties to the FCTC and other countries can use this work as a resource for policy decisions on prioritizing, designing and implementing their tobacco control programs. Based on this overview, governments should focus their efforts on enacting smoke-free legislation and taxing tobacco products. Other tobacco control policies should then be considered to build on their effectiveness and establish comprehensive programs.

## Additional file

Additional file 1:
**Summary of key findings from 59 systematic reviews.**

## Footnotes

1 The exact number of primary studies could not be calculated because some reviews did not report the number of studies they summarized. The estimate of 1150 primary studies is conservative.

2 Evidence from systematic reviews on the effects of providing telephone counselling and cessation advice from healthcare workers was not included due to the clinical context in which these interventions were implemented. This overview focuses on evidence from evaluations of government tobacco control policies.
