# Supplementary material for: Overview of systematic reviews on the health-related effects of government tobacco control policies
Source: BMC Public Health. 2015 Aug 5;15:744. doi: 10.1186/s12889-015-2041-6 (PMC4526291; doi:10.1186/s12889-015-2041-6)
Supplement: Additional file 1: — Summary of key findings from 59 systematic reviews. [file 12889_2015_2041_MOESM1_ESM.docx]

**Summary of Key Findings from 59 Systematic Reviews**

| **MPOWER** | **Review** | **Studies** | **Last search** | **Key findings** | **Quality rating** |
| --- | --- | --- | --- | --- | --- |
| **Protect people from tobacco smoke** | Eriksen & Gottlieb 1998 | 81 | 1994 | - Workplace smoke-free policies reduce cigarette consumption by individual employees in the workplace; their effects on overall consumption, prevalence and cessation are less clear | 6/9 (Authors) |
|  | Hopkins *et al.* 2001 | 16 | 2000 | - Nine studies on smoking behaviour reported reduced tobacco use (median = -1.2 cigarettes/day, range = -4.3 to no change) and increased smoking cessation with smoking bans or restrictions; inconsistent evidence was found for smoking prevalence (range = -11.4 to 1.0) - Nine of ten studies on secondhand smoke (SHS) reported reduced exposure in workplaces with smoking bans or restrictions; four studies evaluated environmental measurements of SHS exposure (median relative percentage difference = -72%, range = -97 to -44) and six studies measured self-reported SHS exposure (median relative percentage difference = -60%, range = -94 to 4) | 10/10 (HE) |
|  | Fichtenberg & Glantz 2002a | 26 | ? | - Smoke-free workplaces reduce smoking prevalence by 3.8% (95% CI = -4.7 to -2.8) and cigarette consumption by 3.1 cigarettes/day/smoker (95% CI = -3.8 to -2.4) - The combined effects of increased smoking cessation and reduced consumption decrease total cigarette consumption per employee by 29%; to achieve this same reduction by cigarette taxation, a proportionately larger cigarette price increase (73%) would be required - Local and state legislation restricting smoking in public spaces and in workplaces reduces smoking prevalence and cigarette consumption | 5/10 (HE) |
|  | Levy & Friend 2003 | 42 | ? | - Workplace smoking restrictions reduce smoking prevalence and cigarette consumption in worksites, but their effect on smoking cessation is less consistent - These findings were supported by population-based studies of workplace smoking restrictions, with quantity consumed reduced by 10-15%, smoking prevalence reduced by 15-20%, and smoking cessation increased by 10-15% - Public clean air laws reduce smoking prevalence and cigarette consumption, and increase smoking cessation rates of the entire population | 6/10 (HE) |
|  | Greaves *et al.* 2006 | 30 | 2004 | - Workplace smoking bans may reduce smoking prevalence and initiation, with complete bans having a stronger effect than partial bans - Self-imposed home smoking bans for adult smokers not only reduces cigarette consumption among adults, but also prevents smoking initiation in adolescents - Inconsistent evidence was found for the effect of school smoking bans on smoking behaviour | 3/10 (HE) |
|  | Thomas *et al.* 2008 | 17 | 2006 | - Workplace smoking bans may be more effective for staff in higher occupational grades - Insufficient evidence exists for the effect of workplace and public smoking bans on other social inequalities in smoking - School bans may be more effective in girls, middle school students, and non-Hispanic students | 5/10 (HE) |
|  | Hopkins *et al.* 2010 | 37 | 2005 | - Workplace and community smoke-free policies reduce tobacco use in various working settings - Policies were found to decrease smoking prevalence (median = −3.4%, IQR = −6.3 to −1.4), increase successful cessation from tobacco use (median = 6.4%, IQR = 2.0 to 9.7), increase quit attempts (median = 4.1%, IQR = −0.7 to 6.8) and decrease quantity of tobacco consumption (median = –2.2 cigarettes/day, IQR = −3.3 to −1.7) | 9/10 (HE) |
|  | Callinan *et al.* 2010 | 50 | 2009 | - 31 studies on SHS, of which 19 were validated with biomarkers, reported reduced SHS exposure in workplaces, restaurants, bars and public places, and no change in exposure in cars or in the home following implementation of a legislative smoking ban - 23 studies on active smoking found limited evidence for legislative smoking bans in reducing smoking prevalence; measures of smoking were studied as co-variables rather than as end-points - 25 studies on health outcomes reported reduced respiratory symptoms, sensory symptoms (especially relating to eye, nose, throat) and hospital admissions for acute myocardial infarction | 7/10 (HE), 10/10 (HSE) |
|  | Mackay *et al.* 2010 | 17 | ? | - Smoke-free legislation reduces acute coronary events (relative risk = 0.90, 95% CI = 0.86 to 0.94) - Studies with longer periods of data collection following implementation of legislation had greater estimates of risk reduction, indicating the effect of smoke-free legislation increases over time | 6/10 (HE) |
|  | Hahn 2010 | 30 | 2010 | - Of the 16 studies on smoking prevalence and/or cigarette consumption, 14 reported reductions with smoke-free policies; these decreases may not be sustained over time - Of the 14 studies on smoking cessation, seven reported increases in quit attempts and/or cessation | 2/10 (HE) |
|  | Kabir *et al.* 2010 | 19 | 2010 | - Comprehensive workplace and public smoke-free policies reduce SHS exposure in children 0 to 17 years and may reduce pediatric health events (i.e., preterm births, emergency asthma admissions) - Home smoke-free policies reduce SHS exposure in children 0 to 17 years by 20-50% | 3/9 (Authors) |
|  | Wilson *et al.* 2012 | 29 | 2009, 2012 | - Smoking bans in public spaces reduce smoking prevalence, with the change in prevalence ranging from -7.4% to -31.9% compared with control groups after 1 to 3.5 years - Inconsistent or insignificant results were found for their effect on smoking initiation and cessation | 4/10 (HE) |
|  | Mozaffarian *et al.* 2012 | 52 | 2011 | - Six studies on community smoking bans reported reduced cardiovascular and respiratory events - 15 studies on workplace smoke-free policies reported increased smoking cessation, reduced cigarette consumption and decreased SHS exposure with both full and partial policies - 18 studies on school-based smoking restrictions reported mixed evidence on smoking behaviour - 13 studies on residence-based smoking restrictions reported reduced smoking prevalence, reduced cigarette consumption, increased rate of cessation attempts, reduced rate of relapse, reduced experimentation in children and decreased SHS exposure in children | 6/10 (HE) |
|  | Tan & Glantz 2012 | 43 | 2011 | - Comprehensive smoke-free legislation (workplaces, restaurants, bars) reduces risk of hospital admissions for coronary events (RR = 0.85, 95% CI = 0.82 to 0.88), other heart diseases (RR = 0.61, 95% CI = 0.44 to 0.85), cerebrovascular accidents (RR = 0.81, 95% CI = 0.70 to 0.94) and respiratory diseases (RR = 0.76, 95% CI = 0.68 to 0.85) - More comprehensive laws are associated with larger reductions in hospital admission risk | 5/11 (Authors) |
|  | CPSTF 2012c | 82 | 2011 | - This update to Callinan *et al*. 2010 found that smoke-free policies: - Reduce self-reported SHS exposure by 50% (IQR = -60 to -43) and biomarker-validated SHS exposure by 50% (IQR = -79 to -12) - Reduce prevalence of tobacco use by 2.7% in adults (IQR = -4.7 to -1.5) - Reduce prevalence of tobacco use in young people (median OR = 0.85, IQR = 0.68 to 0.93) - Increase cessation by 3.8% (IQR = 2 to 17.4) - Reduce tobacco consumption by 1.2 cigarettes/day (range = -3.6 to 0) - Reduce hospital admissions for cardiovascular events (median relative reduction = 5.1%, IQR = -11.6 to -2.2) and asthma (median relative reduction = 20.1%, IQR = -22.0 to -1.3) | 2/10 (Authors) |
|  | Lin *et al.* 2013 | 18 | 2011 | - Smoke-free legislation reduces occurrence of acute myocardial infarction (RR = 0.87, 95% CI = 0.84 to 0.91), with greater reductions in populations with larger decreases in smoking prevalence - Indicates that protective effect of legislation may be caused by decrease in smoking behaviour | 5/10 (HE) |
|  | Kohler & Minkner 2014 | 71 | 2013 | - In a study of state smoke-free policies in Germany, there was no correlation between smoking prevalence and either the maximum fine for violating a smoking ban (Spearman’s ρ = -0.01, p = 0.99) or the maximum fine for failing to enforce a smoking ban (Spearman’s ρ = -0.14, p = 0.61) - Earlier ratification of a smoking ban was correlated with greater reduction in smoking prevalence (Spearman’s ρ = 0.51, p = 0.04) | 5/10 (HE) |
|  | Been *et al.* 2014 | 11 | 2013 | - Smoke-free legislation is associated with reductions in preterm births (-10.4%, 95% CI = -18.8 to -2.0) and hospital admissions for asthma (-10.1%, 95% CI = -15.2 to -5.0) - No association was found with low birth weight (-1.7%, 95% CI = -5.1 to 1.6) | 11/11 (Authors) |
| **Offer help to quit tobacco use** | Hopkins *et al.* 2001 | 5 | 2000 | - Programs to reduce out-of-pocket expenses for smoking cessation therapies increase use of the therapies and levels of smoking cessation | 10/10 (HE) |
|  | Cahill & Perera 2008 | 5 | 2007 | - Three studies on quit and win contests to promote smoking cessation reported 8 to 20% higher quit rates for the quit and win group than for the control group after 12 months - However, the estimated effect of quit and win contests on community-level smoking rates is low, with fewer than one in 500 smokers quitting due to such contests | 10/10 (HE), 9/10 (HSE) |
|  | Leeks *et al.* 2010 | 14 | 2009 | - Workplace financial incentives and competitions implemented with additional interventions (e.g., counseling groups, telephone counseling, smoke-free policies) increase self-reported smoking cessation (median = 4.4%, IQR = 2.7 to 9.4) - Insufficient evidence exists for workplace financial incentives and competitions, when implemented alone, in reducing smoking behaviour | 6/10 (HE) |
|  | Papadakis *et al.* 2010 | 1 | 2009 | - Provider incentives (i.e., financial incentives for providers of smoking cessation interventions) did not promote smoking abstinence (OR = 1.21, 95% CI = 0.98 to 1.5) | 7/10 (HE) |
|  | Cahill & Perera 2011 | 19 | 2010 | - Financial interventions to reward smokers for cessation and continued abstinence, including contests, competitions and fixed payments, do not increase long-term quit rates - The one study that reported improved cessation rates involved significant financial payments for successful abstinence, an approach that is likely only feasible with independently-funded programs | 8/10 (HE), 9/11 (HSE) |
|  | Reda *et al.* 2012 | 11 | 2012 | - Patient-directed financial interventions that fully pay for smoking cessation treatments have positive effects on abstinence, number of patients making quit attempts, and use of the cessation treatments - Financial assistance directed at healthcare providers had no effect on smoking abstinence | 9/10 (HE), 9/11 (HSE), 9/11 (RC) |
|  | CPSTF 2012a | 18 | 2012 | - This update to Hopkins *et al.* 2001 found policies and programs to reduce the financial cost of smoking cessation interventions for smokers: - Increase quit rates by 4.3% (IQR = 0.2 to 6.0) - Increase quite attempt rates by 2.8% (IQR = -0.6 to 9.1) - Two studies found financial assistance interventions reduce prevalence of tobacco use | 2/10 (Authors) |
|  | Johnston *et al.* 2012 | 7 | 2012 | - All five studies included in the meta-analysis are trials of the Smokefree Class Competition, implemented widely across Europe, in which school classes where 90% or more of students refrained from smoking for six months are entered into a competition - Incentives did not prevent smoking initiation in children and adolescents: the pooled relative risk for smoking in the three RCTs was 1.00 (95% CI = 0.84 to 1.19) and the pooled relative risk in the two non-randomized trials was 0.81 (95% CI = 0.61 to 1.08) | 10/10 (HE), 11/11 (HSE) |
|  | Isensee & Hanewinkel 2012 | 5 | 2011 | - The Smokefree Class Competition appears to be effective in preventing smoking, as students participating in the competition were less likely to be smoking at follow-up compared to non-participants (OR = 0.86, 95% CI = 0.79 to 0.94) | 7/10 (HE) |
|  | Hamilton *et al.* 2013 | 18 | 2011 | - Although financial incentives to healthcare providers improve recording of smoking status and frequency of cessation advice, their effect on quit rates and smoking prevalence is inconclusive | 6/10 (HE) |
|  | Likis *et al.* 2014 | 59 |  | - Two RCTs on financial incentives to promote smoking cessation in pregnant women reported greater levels of biologically confirmed smoking cessation in women receiving smoking-contingent incentives - A meta-analysis of 23 RCTs on multicomponent programs reported that incentives were the component most strongly associated with smoking cessation (OR = 3.23; 95% CI = 1.98 to 4.59) | 9/10 (HE) |
|  | Giles *et al.* 2014 | 10 | 2012 | - Financial incentives directed at adult smokers promote smoking cessation - Studies with ≤6 months follow-up (RR = 2.48, 95% CI = 1.77 to 3.46) reported a greater effect than studies with >6 months follow-up (RR = 1.50, 95% CI = 1.05 to 2.14), indicating that financial incentives are more effective for short-term cessation | 10/10 (HE) |
|  | Cahill & Lancaster 2014 | 6 | 2013 | - Five studies of workplace financial incentives reported increased smoking cessation in employees (OR = 1.60, 95% CI = 1.12 to 2.30); however, one study accounted for all of the statistical significance - The one study that could not be included in the meta-analysis reported no change in cessation rates | 8/10 (HE) |
| **Warn about the dangers of tobacco** | Goldman & Glantz 1998 | ? | ? | - Anti-smoking advertisements and mass media campaigns reduce cigarette consumption, but their independent effects could not be separated from concurrent interventions - Mass media campaigns appear to be important components of larger tobacco control programs | 4/10 (HE) |
|  | Willemsen & de Zwart 1999 | ? | 1998 | - Insufficient evidence exists for the effect of warning labels, plain packaging and bans on small packaging (i.e., “kiddie” packs with reduced number of cigarettes) on smoking behaviour - Insufficient evidence exists for the effect of mass media campaigns on smoking behaviour; campaigns may be effective if combined with school smoking prevention programs | 2/10 (HE) |
|  | Lantz *et al.* 2000 | ? | ? | - Mass media campaigns may reduce smoking behaviour in youths, but their individual impact is difficult to evaluate and has not yet been demonstrated - Sustained multi-year campaigns using a strong social marketing approach are likely most effective | 1/10 (Authors) |
|  | Hopkins *et al.* 2001 | 37 | 2000 | - Mass media campaigns reduce smoking prevalence and promote smoking cessation, but only when combined with other interventions; their individual effects cannot be determined - Insufficient evidence exists for mass media series and contests | 10/10 (HE) |
|  | Silver 2001 | 3 | ? | - One study reported that younger adolescents (12 to 13 years) exposed to television anti-smoking advertisements were less likely to become established smokers; the effect was not observed in older adolescents (14 – 15 years) - One study reported that school-based interventions were 40% more effective in reducing the weekly smoking rate of adolescent girls at risk of smoking if they included mass media interventions - The last study did not assess smoking behaviour-related outcomes | 4/10 (HE) |
|  | Friend & Levy 2002 | ? | ? | - Based on studies of statewide tobacco control programs in the US, well-funded and implemented mass media campaigns accompanied by comprehensive tobacco control programs reduce smoking prevalence and cigarette consumption; the effect of campaigns directed at youths is less clear | 4/10 (HE) |
|  | Snyder *et al.* 2004 | 17 | 1998 | - Health communication campaigns cause small but significant changes in health behaviours, with addictive behaviours (i.e., smoking) being more difficult to change than non-addictive behaviours - The 18 studies on smoking campaigns reported a smaller effect size (average correlation = 0.05) than campaigns targeting other health behaviours (average correlation = 0.12) - The 13 studies specifically on smoking cessation campaigns also reported a smaller effect size (average correlation = 0.04) than other cessation campaigns (average correlation = 0.10) | 4/10 (HE) |
|  | Jepson *et al.* 2006 | 44 | 2006 | - Multi-channel mass media interventions, those that combine two or more forms of media (e.g., internet, billboards, newspapers) appear to decrease smoking behavior and increase cessation - Developing culturally appropriate materials that target specific ethnicities and communities or airing campaigns at appropriate times during the day can be effective strategies - There is limited evidence on the different effects of campaigns on population subgroups | 8/10 (HE) |
|  | Niederdeppe *et al.* 2008 | 50 | ? | - Smoking cessation media campaigns are often less effective in persons of low socioeconomic status - Of 18 media campaigns directed at the general population, nine were less effective in persons of lower socioeconomic status than in persons of higher status, six were equally effective, and three were more effective - Of 13 media campaigns directed at persons of low socioeconomic status, eight reported mixed evidence while the other five reported strong evidence for ineffectiveness | 4/9 (Authors) |
|  | Thomas *et al.* 2008 | 5 | 2006 | - Health warnings and labels on tobacco products do not change smoking behavior and smoking-related attitudes in young people - Insufficient evidence exists for packaging interventions on other social inequalities in health | 5/10 (HE) |
|  | Oncken *et al.* 2010 | 8 | 2009 | - Mass media campaigns combined with other interventions reduce smoking initiation, encourage smoking cessation and reduce cigarette consumption - Studies in Indian communities show that media campaigns combined with household interviews, clinical oral exams, and cessation advice reduce tobacco use in low- and middle-income countries - Warning labels on tobacco packages are effective as a first step in encouraging cessation | 2/10 (HE) |
|  | Hammond 2011 | 94 | 2011 | - Large text and pictorial warnings reduce tobacco use and increase quitting likelihood, motivation to quit, likelihood of abstinence after quitting and use of smoking cessation tools - Packaging interventions may also discourage smoking initiation | 5/10 (HE) |
|  | Brinn *et al.* 2012 | 7 | 2010 | - Some evidence suggests that mass media campaigns can prevent smoking initiation in young people (<25 years), but the evidence is not strong and contains methodological flaws - Of seven included studies, three reported that mass media campaigns were associated with reductions in young people’s smoking behaviour, while the other four found no effect | 9/10 (HE) |
|  | Durkin *et al.* 2012 | 29 | 2011 | - Mass media campaigns as part of comprehensive multicomponent tobacco control programs promote smoking cessation and decrease smoking prevalence - The reach, intensity, duration, channel of delivery and message type of the campaign are important; specifically, they should be wide in reach, high in intensity and duration, have television as a primary channel, and include messages on the negative health effects of smoking | 7/10 (HE) |
|  | Wilson *et al.* 2012 | 23 | 2009, 2012 | - Mass media campaigns may reduce smoking behaviour, with stronger evidence for preventing smoking initiation and reducing smoking prevalence; their effect on smoking cessation is less clear - Insufficient evidence exists for the effect of health warning labels on smoking behaviour | 4/10 (HE) |
|  | Guillaumier *et al.* 2012 | 17 | 2012 | - Limited evidence exists on the effect of mass media campaigns in preventing smoking among persons of low socioeconomic status and socially disadvantaged groups | 7/10 (HE), 5/10 (HSE) |
|  | Mozaffarian *et al.* 2012 | 23 | 2011 | - 14 studies on warning labels reported increased awareness of the health risks from smoking, decreased attractiveness of advertisements and packages and reduced cigarette use - Long-term effects of warning labels in reducing smoking behaviour are not known - Nine studies on anti-tobacco media and educational campaigns reported negative attitudes toward smoking, reduced smoking initiation among youths and increased smoking cessation; however, independent effects of campaigns were not examined - Media campaigns are most effective when sustained and combined with other interventions | 6/10 (HE) |
|  | CPSTF 2013 | 64 | 2012 | - Mass-reach health communication interventions that use television as the primary medium: - Reduce prevalence of tobacco use by 5.0% in adults (range = -5.2 to -1.9) and 3.4% in young people (range = -5.3 to -1.6) - Increase cessation by 3.5% (range = 2.0 to 5.0) - Decrease initiation by 6.7% in young people (95% CI = -13.0 to -0.4) - A previous review by the task force (search period 1980 to 2000) also reported reductions in smoking prevalence and tobacco consumption, and increases in cessation | 3/10 (Authors) |
|  | Bala *et al.* 2013 | 11 | 2007 | - Mass media campaigns as part of tobacco control programs may change smoking behaviour in adults; however, included studies were heterogeneous and of mixed methodological quality - Of nine studies on smoking prevalence, two reported significant decreases in prevalence with mass media campaigns while three reported moderate effects - Three of seven studies on tobacco consumption reported decreases - Four of seven studies on quit rates reported moderate to significant increases | 9/10 (HE) |
| **Enforce bans on tobacco advertising, promotion and sponsorship** | Willemsen & de Zwart 1999 | ? | 1998 | - Advertising bans may decrease tobacco consumption, but only in combination with other interventions (e.g., mass media campaigns, educational activities) - Insufficient evidence exists for the independent effect of advertising bans | 2/10 (HE) |
|  | Lantz *et al.* 2000 | ? | ? | - Inconclusive evidence exists for the effect of cigarette advertising bans on youth smoking behaviour | 1/10 (Authors) |
|  | Quentin *et al.* 2007 | 24 | 2004 | - Ten of the 24 studies found advertising bans significantly and negatively influenced tobacco consumption, but the effect size was often narrow - Eight studies found insignificant negative effects | 5/10 (HE) |
|  | Capella *et al.* 2008 | 27 | ? | - Tobacco advertising bans do not reduce cigarette consumption in mature markets (i.e., in the later stages of the product life cycle) - Full advertising bans are no more effective than partial bans in reducing tobacco consumption | 6/10 (HE) |
|  | Thomas *et al.* 2008 | 2 | 2006 | - The effects of tobacco advertising restrictions do not differ by gender or age - Insufficient evidence exists for their effects on other social inequalities in smoking | 5/10 (HE) |
|  | Oncken *et al.* 2010 | 1 | 2009 | - One review on tobacco advertising bans in different countries found that comprehensive bans reduce cigarette consumption, but partial bans are not effective | 2/10 (HE) |
|  | Wilson *et al.* 2012 | 5 | 2009, 2012 | - Insufficient evidence exists for the effect of advertising bans on smoking behaviour | 4/10 (HE) |
|  | Mozaffarian *et al.* 2012 | 6 | 2011 | - Based on well-established relationships between exposure to tobacco advertisements and cigarette use, limiting such advertising likely reduces their pro-tobacco effects - Since advertising restrictions are largely implemented as part of multicomponent strategies, their independent effect cannot be quantified | 6/10 (HE) |
| **Raise taxes on tobacco** | Willemsen & de Zwart 1999 | ? | 1998 | - Increasing the price of tobacco products is the single most effective tobacco control measure - A 10% increase in price reduces tobacco consumption among adolescents by 15 to 20%, and reduces smoking prevalence by at least 6% | 2/10 (HE) |
|  | Lantz *et al.* 2000 | ? | ? | - Increasing the price of tobacco likely reduces youth smoking behaviour - Youths are likely more responsive than adults to changes in cigarette prices due to the addictive nature of cigarettes: youths, who have been smoking for a relatively shorter time, are more likely to alter their smoking behaviour than adults, who have been smoking for longer | 1/10 (Authors) |
|  | Wakefield & Chaloupka 2000 | ? | 1999 | - Based on surveys of five statewide tobacco control programs in the US, tobacco taxation decreases tobacco consumption in adolescents and among all citizens - The effect of taxation is strengthened if it is combined with other tobacco control interventions | 4/10 (HE) |
|  | Hopkins *et al.* 2001 | 25 | 2000 | - Increasing tobacco price reduces smoking prevalence and cigarette consumption, and promotes smoking cessation, with a median price elasticities of -0.37 (range = not statistically significant to -1.19) and -0.23 (range = -0.68 to 0) for prevalence and consumption, respectively | 10/10 (HE) |
|  | Gallet & List 2003 | 86 | ? | - A meta-analysis of price elasticities of demand found increases in tobacco price reduces cigarette consumption, with demand more responsive to price in the long-run - Teens are more sensitive to price, and more recent studies tend to report more inelastic estimates | 1/11 (Authors) |
|  | Greaves *et al.* 2006 | 18 | 2004 | - Increasing tobacco price through taxation significantly reduces smoking behaviour - Among adolescents, tax increases reduce smoking initiation and cigarette consumption; the effect on smoking cessation is not clear | 3/10 (HE) |
|  | Laporte 2006 | 36 | ? | - A meta-analysis of price-elasticities of demand reported an average elasticity of -0.69, indicating a 6.9% decrease in cigarette consumption for every 10% increase in price | 1/11 (Authors) |
|  | Thomas *et al.* 2008 | 42 | 2006 | - Increasing tobacco price has a greater effect on adults with low incomes or manual occupations - Price increases are effective in reducing youth smoking, with the effect potentially stronger in older youths, boys and black or Hispanic adolescents | 5/10 (HE) |
|  | Oncken *et al.* 2010 | 12 | 2009 | - Increasing tobacco price reduces cigarette consumption in both high-income and low- and middle-income countries, and may have a greater impact in low- and middle-income countries - Price increases reduce smoking in pregnancy and deaths due to sudden infant death syndrome | 2/10 (HE) |
|  | Rice *et al.* 2010 | 45 | 2007 | - Price increases are effective in reducing smoking behaviour among young people - Two longitudinal studies on smoking participation (i.e., likelihood of smoking) reported an average price elasticity of -0.176; seven repeated cross-sectional studies reported an average elasticity of -0.489 (range = -0.77 to -0.126) - All three studies on smoking prevalence reported reductions in prevalence with increased tobacco price, with the strongest study finding price elasticities between -0.131 and -0.243 - For consumption by individual smokers, one longitudinal study reported a price elasticity of -0.731; five repeated cross-sectional studies found an average elasticity of -0.327 (range = -0.02 to -0.561) - For total consumption (i.e., quantity of cigarettes smoked by an entire population), one longitudinal study reported a price elasticity of -0.844, while five repeated cross-sectional studies found an average elasticity of -0.365 (range = -1.148 to 0.86) - Increasing tobacco price deters young people from initiating smoking, with an average price elasticity of -0.597 (range = -0.111 to -0.912) reported across five longitudinal studies - Increasing tobacco price encourages young people to quit smoking, but the effect is moderate for encouraging sustained smoking cessation | 7/10 (Authors) |
|  | Bader *et al.* 2011 | 108 | 2010 | - Increasing tobacco price decreases smoking prevalence and cigarette consumption and promotes smoking cessation in youths (<19 years) and young adults (18 – 24 years) - Evidence on smoking initiation in these two groups is inconclusive - Increasing price reduces smoking behaviour in persons of low socioeconomic status - Insufficient evidence exists for impacts on heavy/long-term smokers, persons with dual diagnoses (i.e., with concomitant mental health disorders or substance abuse disorders) and Aboriginals | 5/9 (HSE) |
|  | IARC 2011 | 90 | 2010 | - Increasing tobacco price decreases cigarette consumption in countries at all levels of income - Price elasticities of cigarette consumption appear to be more elastic in low-income countries - Increasing tobacco price decreases smoking prevalence and cigarette consumption in individual smokers, with the price elasticity of prevalence ranging from -0.6 to -0.2 - Increasing tobacco price decreases smoking prevalence, cigarette consumption, and smoking initiation in young people, and young people appear to be more price sensitive than adults - People of low socioeconomic status are more sensitive to tobacco price in high-income countries, while the relationship in middle- and low-income countries is mixed - Increasing tobacco price decreases premature deaths and morbidities attributable to smoking | 2/10 (Authors) |
|  | Mozaffarian *et al.* 2012 | 5 | 2011 | - Strong evidence exists for tobacco taxation in reducing smoking behaviour, particularly in youths | 6/10 (HE) |
|  | Wilson *et al.* 2012 | 36 | 2009, 2012 | - Tobacco taxation decreases smoking behaviour and has a stronger impact on youths - Strong evidence exists for reducing smoking prevalence and moderate evidence exists for preventing smoking initiation and increasing smoking cessation - Every 10% increase in price decreases smoking initiation by 0.9 to 6.5%, increases cessation by 3.75 to 11.7%, and decreases prevalence by 1 to 14.1% in youths and up to 4.5% in adults. | 4/10 (HE) |
|  | CPSTF 2012b | 116 | 2012 | - This update to IARC 2011 and Wilson *et al.* 2012 found that a 10% increase in tobacco price: - Reduces total demand of tobacco products by 3.7% in adults (IQR = -4.7 to -2.9) and 7.4% in young people (i.e., <30 years of age) (IQR = -11.3 to -5.7) - Reduces prevalence of tobacco use by 1.8% in adults (IQR = -3.1 to -1.1) and 3.6% in young people (IQR = -7.3 to -2.4) - Increases cessation by 3.75% in adults and 9.3% in young people (IQR = 3.7 to 10) - Reduces initiation by 4.3% in young people (IQR = -9.0 to 0) - Included studies that did not evaluate price elasticities reported similar findings, as well as reductions in mortality rates from lung cancer and cardiovascular disease - Price increases may be more effective in low-income tobacco users and Hispanics | 3/10 (Authors) |
|  | Guindon 2013 | 27 | 2012 | - The existing evidence on the effect of tobacco price increases or taxation on smoking onset (i.e., transition between never smoking and smoking) is insufficient due to methodological limitations | 5/10 (Authors) |
| **Restrict sales to minors** | Willemsen & de Zwart 1999 | ? | 1998 | - Insufficient evidence exists for the effect of youth access restrictions on smoking behaviour - Age limits on tobacco sales are unrealistic since full compliance among tobacconists is required, which is difficult to maintain | 2/10 (HE) |
|  | Lantz *et al.* 2000 | ? | ? | - The effect of youth access restrictions on youth smoking behaviour is unclear | 1/10 (Authors) |
|  | Fichtenberg & Glantz 2002b | 8 | 2001 | - Youth access interventions do not affect youth smoking prevalence - Merchant compliance and increases in compliance with youth access laws are not associated with 30-day or regular teen smoking prevalence - Smoking prevalence among youths does not differ between communities with youth access restrictions and communities without such restrictions | 5/10 (HE) |
|  | Stead & Lancaster 2005 | 35 | 2008 | - Youth access interventions can reduce illegal tobacco sales to youths, especially if they involve active enforcement instead of only education - Mixed evidence exists for youth access interventions on youth smoking prevalence and perceptions of ease of access to tobacco | 7/10 (HE) |
|  | Greaves *et al.* 2006 | 24 | 2004 | - Youth access restrictions may reduce the number of successful tobacco purchase attempts by minors, but do not affect the general availability (i.e., from non-commercial sources) of cigarettes - The different effects of sales restrictions in vulnerable populations could not be determined | 3/10 (HE) |
|  | Thomas *et al.* 2008 | 13 | 2006 | - Restrictions on sales to minors may be more effective in reducing cigarette consumption among girls, younger students and Caucasians, but results are not consistent across studies | 5/10 (HE) |
|  | Richardson *et al.* 2009 | 60 | 2007 | - One study found no difference in youth smoking prevalence between communities with youth access restrictions and communities without such restrictions - Another study found that youth access restrictions decrease the likelihood of youths being in higher stages of smoking uptake, and were more effective for youths already in higher stages | 5/10 (HE) |
|  | DiFranza 2012 | 424 | 2010 | - Laws prohibiting the sale of tobacco to minors and merchant education programs will have no impact without effective enforcement - 19 studies evaluating enforcement efforts that successively disrupted the sale of tobacco poducts to minors all reported declines in youth tobacco use - Poorly enforced efforts that fail to disrupt sale to minors do not affect youth smoking behaviour | 5/10 (HE) |

A question mark (“?”) indicates the review did not report the number of studies included or the year of last search. CI = confidence interval; CPSTF = Community Preventive Services Task Force; HE = *Health Evidence*; HSE = *Health Systems Evidence*; IARC = International Agency for Research on Cancer; IQR = interquartile range; OR = odds ratio; RC = *Rx for Change*; RCT = randomized controlled trial; and RR = relative risk.
